# Effects of dietary intervention on vitamin B_12_ status and cognitive level of 18-month-old toddlers in high-poverty areas: a cluster-randomized controlled trial

**DOI:** 10.1186/s12887-019-1716-z

**Published:** 2019-09-13

**Authors:** Xiaoyang Sheng, Junli Wang, Feng Li, Fengxiu Ouyang, Jingqiu Ma

**Affiliations:** 1 Department of Children and Adolescents Health Care, Xin Hua Hospital, School of Medicine, Shanghai Jiao Tong University, Shanghai Institute for Pediatric Research, MOE-Shanghai Key Laboratory of Children’s Environmental Health, No.1665, Kongjiang Road, Yangpu District, Shanghai, 200092 China; 2 Ministry of Education and Shanghai Key Laboratory of Children’s Environmental Health, Xin Hua Hospital, School of Medicine, Shanghai Jiao Tong University, No.1665, Kongjiang Road, Yangpu District, Shanghai, 200092 China; 3 Shanghai Institute for Pediatric Research, Xin Hua Hospital, School of Medicine, Shanghai Jiao Tong University, Shanghai Key Laboratory of Pediatric Gastroenterology and Nutrition, No.1665, Kongjiang Road, Yangpu District, Shanghai, 200092 China

**Keywords:** Vitamin B_12_, Deficiency, Toddler, Complementary food, Neurodevelopment

## Abstract

**Background:**

The local diet in high-poverty areas in China is mainly vegetarian, and children may be more vulnerable to vitamin B_12_ deficiency.

**Objective:**

The aims of this study were to explore the vitamin B_12_ status of toddlers living in high-poverty areas of China and to observe the effects of different complementary foods on the vitamin B_12_ status and cognitive level of these toddlers.

**Methods:**

The study was nested within a cluster-randomized controlled trial implemented in 60 administrative villages (clusters) of Xichou County in which infants aged 6 months old were randomized to receive 50 g/d of pork (meat group), an equi-caloric fortified cereal supplement (fortified cereal group) or local cereal supplement (local cereal group) for one year. At 18 months, a subsample of the 180 toddlers (60 from each group) was randomly tested for serum vitamin B_12_ and total homocysteine (tHcy) levels, and their neurodevelopment was evaluated.

**Results:**

The median serum concentrations of vitamin B_12_ and tHcy were 360.0 pg/mL and 8.2 μmol/L, respectively, in children aged 18 months. Serum vitamin B_12_ concentrations less than 300 pg/mL were found in 62 (34.4%) children, and concentrations less than 200 pg/mL were found in 30 (16.7%) children. The median vitamin B_12_ concentration was significantly different among the three groups (*P* < 0.001). The highest vitamin B_12_ level was demonstrated in the fortified cereal group (509.5 pg/mL), followed by the meat group (338.0 pg/mL) and the local cereal group (241.0 pg/mL). Vitamin B_12_ concentration was positively correlated with the cognitive score (*P* < 0.001) and the fine motor score (*P* = 0.023) of the Bayley Scales of Infant Development, 3rd Edition (BSID III) screening test. Compared to the local cereal group, children in the meat group had higher cognitive scores (*P* < 0.05).

**Conclusion:**

In poor rural areas of China, vitamin B_12_ deficiency in toddlers was common due to low dietary vitamin B_12_ intake. Fortified cereal and meat could help improve the vitamin B_12_ status of children and might improve their cognitive levels.

**Trial registration:**

The larger trial in which this study was nested was registered at clinical trials.gov as NCT00726102. It was registered on July 31, 2008.

## Background

Vitamin B_12_, also called cobalamin, is an essential water-soluble micronutrient found exclusively in animal-derived foods, such as meat, eggs, fish and milk, and it cannot be synthesized by the body. Vitamin B_12_ deficiency can cause hematological shortages, resulting in increased red cell mean corpuscular volume (MCV) and macrocytic anemia through the alteration of erythropoiesis [1]. Furthermore, vitamin B_12_ is necessary for the development and initial myelination of the central nervous system as well as for the maintenance of its normal function [2].

Vitamin B_12_ deficiency is a worldwide public health issue. People who consume a vegetarian diet or limit animal products may develop vitamin B_12_ deficiency. The prevalence of low Vitamin B_12_ status is high in low-income settings, especially in rapidly growing children with a high demand for vitamin B_12_. In North India, one-third of children aged 6 to 35 months had a plasma vitamin B_12_ concentration ≤ 200 pmol/L [3]. Recently, a cross-sectional household cluster survey revealed that 30.2% of infants and toddlers aged 6 to 23 months in two districts in Nepal had a vitamin B_12_ deficiency (serum vitamin B_12_ < 150 pmol/L) [4]. Another community-based, randomized, double-blind clinical trial in Nepal demonstrated that more than 50% of breastfed infants aged 6 to 11 months with a length-for-age z-score (LAZ) < − 1 have a vitamin B_12_ deficiency [5].

It is also known that vitamin B_12_ deficiency is one potential cause of adverse developmental outcomes. In recent years, neurodevelopment in children has been linked with vitamin B_12_ status in several studies. Recently, the vitamin B_12_ status of Nepalese infants showed positive associations with development and performance on social perception tasks and visuospatial abilities at 5 years of age [6]. Indian infants aged 12–18 months with a vitamin B_12_ deficiency presented with lower psychomotor and mental development scores compared with the scores of infants with higher vitamin B_12_ status [7].

To date, there have been no reports on the vitamin B_12_ status of infants and young children in China, especially in poor areas. Due to low socioeconomic status, the local diet is mainly vegetarian. Therefore, infants and toddlers may be more vulnerable to vitamin B_12_ deficiency. Beginning in March 2009, a large intervention trial was conducted in a poor rural area, Xichou County, located in Yunnan Province, China, in which the effect of meat was evaluated as the primary complementary food affecting the linear growth of toddlers between 6 and 18 months of age. In comparison, equi-caloric quantities of rice cereal or of micronutrient-fortified rice cereal were used as controls. Our substudy was a cross-sectional subsample nested within a large intervention trial.

In this substudy, our objectives were to determine 1) the levels of serum vitamin B_12_, total homocysteine (tHcy) and hemoglobin in toddlers at 18 months of age; 2) the relationship between cognition and motor development and vitamin B_12_ nutritional status of toddlers at 18 months of age; and 3) the efficacy of complementary foods, including micronutrient-fortified rice cereal and red meat, for improving vitamin B_12_ status.

## Methods

### Study design

The substudy was nested within a cluster-randomized, nonmasked, controlled efficacy trial conducted from March 2009 to December 2011 in a poor rural area, Xichou County, located in the Yunnan Province of China. Details of the study design have been described elsewhere [8, 9].

In the study areas, early complementary foods offered to infants are mainly various types of plant-based gruels made from cereal grains or starchy roots and tubers. Sixty administrative villages (clusters) in 9 districts in Xichou County were included in this study. Six-month-old infants were randomized to receive 50 g/d of pork (meat group), an equi-caloric micronutrient-fortified rice cereal-based supplement (fortified cereal group), or a local nonfortified rice cereal supplement (local cereal group) for one year [8, 9]. Approximately 20–30 infants were involved in each administrative village. Infants were identified at 3–5 months of age by their community doctors. Eligibility criteria for infants included term delivery without serious neonatal complications, absence of acute or chronic illness, healthy singleton status with birth weight > 2000 g, no metabolic or physical problems and being exclusively breastfed [8, 9].

Administering the intervention to participants and evaluating the compliance and morbidity of participants were performed by the specially trained community doctors in each of the small communities within each village. The fresh certified-safe lean pork was purchased weekly and was minced and accurately weighed into daily 50 g (80 kcal) aliquots containing 0.21 μg vitamin B_12_ and stored frozen until transported weekly to the district hospitals which were serving the meat group villages [8, 9]. The quantity of either micronutrient-fortified cereal (20 g, 80 kcal) or local cereal (20 g, 80 kcal) was designed to be equi-caloric to the daily supply of pork. The participants in the fortified cereal group received a commercial product (Nestle, fortified with vitamin B_12_, iron and zinc) and the vitamin B_12_ content in 20 g of the fortified cereal was 0.2 μg. Local rice cereal was made from a mixture of glutinous rice flour, white granulated sugar and honey, without vitamin B_12_ [8, 9]. Supplies of these control foods were provided weekly to the district hospitals in all participating villages, and the control foods were collected at these hospitals and distributed weekly by the participating community doctors [8, 9]. Participants in the meat group, fortified cereal group and local cereal group were encouraged to consume 50 g of red meat, 20 g of fortified rice cereal and 20 g of local rice cereal each day, respectively.

Furthermore, the participants were seen by the assessment team at the nearest hospital at baseline and at 3-month follow-up intervals. All anthropometric measurements, including length, weight, and head circumference at the age of 6 and 18 months, were measured using standardized procedures by the assessment team. The effect of supplementary food on linear growth and micronutrient status between 6 and 18 months of age was assessed. The operation manual for the study is shown in more detail in Additional file 1.

### Participants

**Fig. 1:**
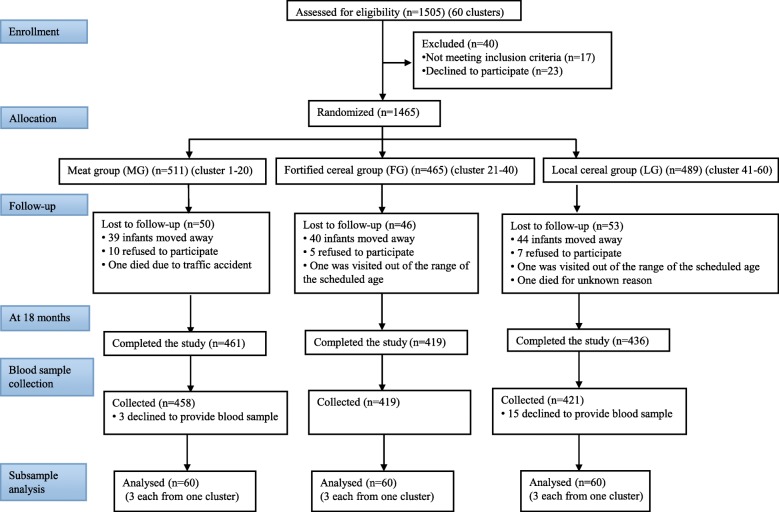
Participant flow through the study

A total of 1465 infants completed the study at baseline (6 months), including 511 in the meat group, 465 in the fortified cereal group and 489 in the local cereal group. At 18 months, a total of 1316 children completed the study, including 461 in the meat group, 419 in the fortified cereal group and 436 in the local cereal group. Three children in the meat group and 15 children in the local cereal group did not provide blood samples. Finally, a total of 1298 children, without upper respiratory tract infection, diarrhea or other acute infections, provided blood samples. The flow of the participants through the study is shown in Fig. 1.

### Blood sampling and biochemical assessment

Hemoglobin (Hb), mean corpuscular volume (MCV), mean corpuscular hemoglobin (MCH) and mean corpuscular hemoglobin concentration (MCHC) were measured in whole blood with an HC3000 auto-hematology analyzer on the day of blood sampling. Peripheral venous blood samples of 5 mL were collected by trained and experienced phlebotomists and put into a trace element-free, additive-free evacuated tube. Serum was divided into two aliquots and stored immediately at − 20 °C; then, they were transported frozen and stored at − 80 °C until serum vitamin B_12_ and tHcy analysis. The individuals who carried out the laboratory analyses were unaware of the participants’ group assignments.

Serum vitamin B_12_ was measured by an automatic ACCESS microparticle chemiluminescence immunoassay analyzer with a chemiluminescence immune assay (Beckman Coulter, Inc., USA). Serum tHcy was measured on a Hitachi 7600-120E automated biochemistry analyzer with enzymatic cycling assay (DiaSys Diagnostic Systems GmbH, Germany).

### Subsample size

According to the vitamin B_12_ levels of children from poor areas reported in the literature [10–12], it is estimated that the average serum vitamin B_12_ concentration of children in this study would be 300 pg/mL in the meat group, 350 pg/mL in the fortified cereal group, and 250 pg/mL in the local cereal group, with the assumption of a standard deviation of 150 pg/mL. Based on the sample size calculation formula for a multisample study design [13], blood samples from 60 children in each group were required to determine significant differences among the three groups and to achieve a power of 90% at a 5% level of significance. A total of 180 blood samples were collected. Sixty was randomly selected from the meat group (cluster 1–20), 60 from the fortified cereal group (cluster 21–40) and 60 from the local cereal group (cluster 41–60) by two investigators who were not involved in the recruitment and data collection, with 3 blood samples from each cluster.

### Neurocognitive testing

The cognitive scale and the fine motor and gross motor subtests of the Bayley Scales of Infant Development, 3rd Edition (BSID III) screening test were used to assess the infants’ development at 18 months of age [14]. The language domain was not performed because it was not applicable in the setting of Yunan, China. All children were assessed within a two-week window at approximately 18 months of age, and the assessments were administered either by a psychologist familiar with the test or by trained assistants/students. All assistants/students were graduate students majoring in pediatrics who received a two-month intensive training on the BSID III test in the Department of Children and Adolescents Health Care of Xin Hua Hospital affiliated to Shanghai Jiao Tong University School of Medicine. Before the children were evaluated with the BSID III test, the evaluators had been living and working locally in rural Yunnan for several months. All evaluators were able to communicate with the participants and their parents in Mandarin and simple local dialects. Community sites were adapted and arranged to provide a standardized environment for the testing. When tests were administered, the mothers were present. If the child did not cooperate during the test, the evaluator would try again when the child was in a better mood. Each test administration took approximately 30 min. Double data entry was adopted, and the original paper copies of the data were retained for reverification.

### Definitions

Xichou County in Yunnan Province has an average elevation of 1400 m above sea level, so anemia was defined as Hb < 115 g/L [9, 15].

According to the World Health Organization (WHO) [12, 16, 17], serum vitamin B_12_ concentrations of < 200 pg/mL, 200–300 pg/mL and > 300 pg/mL were used to classify individuals as deficient, marginally deficient, or adequate, respectively. However, stand-alone marker of serum vitamin B_12_ has been proven insufficient for the unequivocal diagnosis of vitamin B_12_ deficiency [18]. Therefore, a tHcy concentration of > 12 μmol/L was also used to obtain a correct diagnosis, based on the suggestions in the recent literature [1].

The weight-for-age z-score (WAZ), LAZ, weight-for-length z-score (WLZ) and head circumference-for-age z-score (HcAZ) were calculated according to the 2006 WHO Child Growth Standards using WHO Anthro 2011 software [19]. Underweight, stunting, and wasting were defined as WAZ < -2, LAZ < -2, and WLZ < -2, respectively.

### Statistical analysis

All data were analyzed using descriptive and frequency statistics in SPSS 13.0 for Windows. The results are presented as the mean ± SD for normally distributed continuous variables or as the median (interquartile range, IQR) for data not normally distributed. Log transformations were used where residuals were skewed or exhibited nonconstant variance. Anthropometric variables, Hb level and subtest scores of the BSID III screening test were compared among groups by ANOVA. Not normally distributed data, including serum vitamin B_12_ and tHcy concentrations and maternal education duration, were compared after log transformations among groups by ANOVA. The LSD multiple comparison test was used for the post hoc test if the overall ANOVA had a *P* value less than 0.05. Chi-square tests were used for categorical variables. To address the clustering effect, the differences in serum vitamin B_12_ and tHcy concentrations (after log transformations) and cognitive score between the meat or fortified cereal groups and the local cereal group were analyzed using mixed-effect linear models, and the local cereal group was used as the reference. Outcomes were analyzed with villages as the random factor and group as the fixed factor. Comparisons were expressed as estimated differences with 95%CIs. Correlations between serum vitamin B_12_ and tHcy levels and the associations of vitamin B_12_ or tHcy levels with LAZ, WAZ, WLZ, HcAZ, Hb, MCV, MCH and MCHC were tested by Pearson correlation after log transformation. The associations between the subtest scores of the BSID III screening test (the cognitive score, the fine motor score or the gross motor score) and the serum vitamin B_12_ or tHcy concentrations were examined by a linear regression model on condition of the adjusting covariates (maternal education duration and birth weight) with “enter” as the regression method. All statistical tests were two-tailed and *P* values < 0.05 were considered statistically significant.

## Results

### Anthropometric outcomes of the subsample

A total of 180 blood samples, 60 from each group, were selected by a simple random sampling from 1298 blood samples stored at − 80 °C in Shanghai Key Laboratory of Children’s Environmental Health.

The mean age of toddlers in the meat group, fortified cereal group and local cereal group was 17.9 ± 0.1, 18.0 ± 0.2 and 17.9 ± 0.2 months, respectively. The percentages of males among the three groups were 51.7, 48.3 and 48.3%, respectively. The mean birth weights in the three groups were 3075.7 ± 380.2, 3039.6 ± 362.1 and 3005.7 ± 373.4 g, respectively, and the median maternal education duration in the three groups was 9(6–9), 9(6–9) and 9(6–9) years, respectively. There was no significant difference in birth weight and maternal education duration; furthermore, the LAZ, WAZ, WLZ and HcAZ at 18 months of age were similar among the three groups (all *P* > 0.05) (Table 1).

**Table 1:** Comparison of anthropometric outcomes and biochemical markers by intervention group at age 18 months

Variables	All subjects (*n* = 180)	Meat group (*n* = 60)	Fortified cereal group (*n* = 60)	Local cereal group (*n* = 60)	*P* value
Age (months)	17.9 ± 0.2^a)^	17.9 ± 0.1	18.0 ± 0.2	17.9 ± 0.2	0.001
Male [*n*(%)]	91 (50.6)	31 (51.7)	29 (48.3)	29 (48.3)	0.915
LAZ	−1.6 ± 1.0	−1.5 ± 1.0	−1.8 ± 0.9	− 1.6 ± 1.1	0.196
WAZ	−0.9 ± 0.9	−0.9 ± 0.9	−1.0 ± 0.9	−0.8 ± 1.0	0.417
WLZ	−0.2 ± 0.9	−0.3 ± 0.9	− 0.2 ± 0.8	−0.1 ± 0.9	0.361
HcAZ	−0.3 ± 0.9	−0.4 ± 0.8	− 0.3 ± 0.9	−0.3 ± 0.9	0.802
VitB_12_ (pg/mL)^b)^	360.0 (233.0~573.8)^c)^	338.0 (233.0~578.3)^d)e)^	509.5 (364.8~992.3)^d)^	241.0 (185.5~396.3)	< 0.001
tHcy (μmol/L)^b)^	8.2 (6.9~10.2)	8.0 (6.6~10.3)^d)^	7.4 (6.1~8.9)^d)^	9.2 (7.8~11.3)	< 0.001
Hb (g/L)	122.5 ± 11.8	123.3 ± 12.5	123.6 ± 9.9	120.7 ± 12.8	0.346
MCV (fL)	78.9 ± 6.1	79.7 ± 5.0	78.8 ± 6.2	78.3 ± 6.9	0.445
MCH (pg)	26.8 ± 2.5	27.1 ± 2.4	26.9 ± 2.5	26.3 ± 2.6	0.215
MCHC (g/L)	339.6 ± 19.2	340.5 ± 19.9	342.0 ± 21.9	336.5 ± 15.1	0.266

a) Mean ± SD (for all results in this format)

b) Data there were not normally distributed were compared after log transformations among the three groups by ANOVA

c) Median; interquartile range in parentheses (for all results in this format)

d) Compared with the local cereal group, *P* < 0.05

e) Compared with the fortified cereal group, *P* < 0.05

### Vitamin B_12_ status and anemia

The median (IQR) concentration of serum vitamin B_12_ was 360.0 (233.0–573.8) pg/mL in all children at 18 months of age. Deficient (< 200 pg/mL) and marginally deficient (200–300 pg/mL) serum vitamin B_12_ concentrations were found in 62 (34.4%) children and 30 (16.7%) children were vitamin B_12_ deficient.

The median (IQR) level of serum tHcy was 8.2 (6.9–10.2) μmol/L in all children. Serum tHcy > 12 μmol/L was found in 21 (11.7%) children, and among them, 7 children also had serum vitamin B_12_ levels < 200 pg/mL, and another 7 children had serum vitamin B_12_ levels of 200–300 pg/mL.

The mean Hb level in all children was 122.5 ± 11.8 g/L. Anemia (Hb < 115 g/L) was found in 42 (23.3%) children, and among them, 14 children also had serum vitamin B_12_ levels < 300 pg/mL. There was no significant difference in Hb, MCV, MCH and MCHC among the meat group, fortified cereal group and local cereal group (*P* > 0.05) (Table 1).

Pearson correlation results suggested that serum vitamin B_12_ levels were negatively correlated with tHcy after log transformation (*r* = − 0.45, *P* < 0.001). There was no association between vitamin B_12_ level and the LAZ, WAZ, WLZ, HcAZ, Hb, MCV, MCH or MCHC (*P* > 0.05). The tHcy level was positively correlated with the WLZ after log transformation (*r* = 0.15, *P* = 0.039), but there was no correlation between the tHcy level and the LAZ, WAZ, HcAZ, Hb, MCV, MCH or MCHC (*P* > 0.05).

### Effects of different food interventions on vitamin B_12_ status

The median vitamin B_12_ concentration was significantly different among the three groups (*P* < 0.001). The fortified cereal group had the highest level (509.5 pg/ml), followed by the meat group (338.0 pg/ml) and the local cereal group (241.0 pg/mL) (Table 1). Compared with the local cereal group, toddlers in the meat group (estimated difference 0.16, 95% CI: 0.06~0.26, *P* = 0.002) and fortified cereal group (estimated difference 0.35, 95% CI: 0.26~0.45, *P* < 0.001) had higher vitamin B_12_ concentrations, according to the mixed-effect linear models after log transformations.

Among 62 children whose vitamin B_12_ concentration was < 300 pg/mL, there were 22 (36.7%) in the meat group, 3 (5.0%) in the fortified cereal group and 37 (61.7%) in the local cereal group. The difference between the three groups was statistically significant (*χ*^*2*^ = 42.86, *P* < 0.001).

There was a significant difference in the serum tHcy levels among the three groups (*P* < 0.001). The serum tHcy levels in both the meat group and fortified cereal group were lower than the levels in the local cereal group (*P* < 0.05) (Table 1). Compared with the local cereal group, toddlers in the meat group (estimated difference − 0.08, 95% CI: − 0.13~ − 0.02, *P* = 0.005) and fortified cereal group (estimated difference − 0.12, 95% CI: − 0.18~ − 0.07, *P* < 0.001) had lower tHcy concentrations, according to the mixed-effect linear models after log transformations.

Among 21 children whose tHcy level > 12 μmol/L, 7 (11.7%) were in the meat group, 3 (5.0%) were in the fortified cereal group and 11 (18.3%) were in the local cereal group. There was no difference among the three groups (*χ*^*2*^ = 5.18, *P* > 0.05). Serum tHcy > 12 μmol/L and vitamin B_12_ < 300 pg/mL were found in 14 (7.8%) toddlers. Among them, 7 toddlers had a vitamin B_12_ level of < 200 pg/mL.

### Vitamin B_12_ status and neurocognitive development

Because 9 children were unable to cooperate with staff to complete the test, including 2 in the meat group, 5 in the fortified cereal group and 2 the in local cereal group, 171 children completed the BSID III screening test at 18 months of age.

There were significant differences in the cognitive score of the BSID III screening test among the three groups (*P* = 0.020). Compared to the local cereal group, children in both the meat group and fortified cereal group had higher cognitive scores (*P* < 0.05) (Table 2). The mixed-effect linear models showed that the meat group had higher cognitive scores than the local cereal group (estimated difference 0.99, 95% CI: 0.21~1.76, *P* = 0.013). The cognitive score of the fortified cereal group was also higher than that of the local cereal group, but the difference was not statistically significant (estimated difference 0.78, 95% CI: − 0.00~1.57, *P* = 0.051).

**Table 2:** Comparison of the BSID III screening test scores at age 18 months by intervention group

Variables	All subjects (*n* = 171)	Meat group (*n* = 58)	Fortified cereal group (*n* = 55)	Local cereal group (*n* = 58)	Statistics	*P* value
Cognitive score	21.0 ± 2.0^a)^	21.3 ± 1.9^b)^	21.2 ± 2.0^b)^	20.4 ± 2.0	*F* = 4.02	0.020
Fine motor score	18.2 ± 1.1	18.0 ± 1.1	18.3 ± 1.1	18.3 ± 1.0	*F* = 1.63	0.199
Gross motor score	20.1 ± 1.2	20.1 ± 1.4	20.0 ± 1.1	20.2 ± 1.1	*F* = 0.67	0.513

a) Mean ± SD (for all results in this format)

b) Compared with the local cereal group, *P* < 0.05

In 171 children, serum vitamin B_12_ concentrations were positively correlated with the cognitive score (*beta* = 2.15, *SE* = 0.55, *P* < 0.001) and the fine motor score (*beta* = 0.71, *SE* = 0.31, *P* = 0.023), according to a multiple linear regression analysis.

## Discussion

This study is the first to report the vitamin B_12_ status of 18-month-old toddlers in a poor rural area of China. Due to the low economic level, the diet of the local people in the study areas is mainly vegetarian. Vitamin B_12_ content in the local rice-based complementary foods was too low to meet the physiological needs of infants and young children. Therefore, local populations, especially infants and young children, are likely at high risk for vitamin B_12_ deficiency. In the population subsample, serum vitamin B_12_ concentrations < 300 pg/mL were found in 34.4% of toddlers, and concentrations of < 200 pg/mL were found in 16.7% of toddlers. In addition, toddlers with serum vitamin B_12_ concentrations < 300 pg/mL were comprised up to 61.7% of the local cereal group. Our results suggested that the vitamin B_12_ status of toddlers in the study area is poor, similar to that in other developing countries [3–5].

Vitamin B_12_ status was the best in the fortified cereal group, followed by the meat group and local cereal group. Both the daily 50 g of lean pork and 20 g of fortified cereal contained approximately 0.2 μg of vitamin B_12_, and there was no vitamin B_12_ supplement in the local cereal. As a result, during the one-year follow-up, the intake of vitamin B_12_ in the meat group and fortified cereal group should have been higher than that in the local cereal group. In addition, though vitamin B_12_ from fortified cereal is directly consumed, meat may lose some of its vitamin B_12_ during the cooking process. Therefore, the actual vitamin B_12_ intake in toddlers in the fortified cereal group may be higher than that in the meat group, which may explain why the vitamin B_12_ status of toddlers in the fortified cereal group was better than that in the meat group. At present, according to the WHO, the recommended nutrient intake (RNI) for vitamin B_12_ in children aged 0–3 years is as follows: 0.4 μg/d for ages 0–6 months, 0.5 μg/d for ages 7–12 months, and 0.9 μg/d for ages 1–3 years [20]. Therefore, even in the meat group and fortified cereal group, the intake of vitamin B_12_ from the intervention foods after the age of 12 months was still far below the recommended intake, which led to the prevalence of vitamin B_12_ deficiency among toddlers in this study.

Vitamin B_12_ is extremely important for the protection of nerve cells. Vitamin B_12_ deficiency may have adverse outcomes through a variety of metabolic pathways that can alter energy use and reduce the production of neurotransmitters and myelin. Myelin is the main component of white matter in the brain and is essential for nerve conductivity. Disorders in myelination decrease the conduction velocity of multiple systems both in the central and peripheral nervous systems [21]. Therefore, the correlation between vitamin B_12_ status and the neurodevelopment of young children in poor areas deserves our attention. In our study, vitamin B_12_ status was found to be significantly correlated with the cognitive score and the fine motor score of the BSID III screening test, which was similar to the results of other studies [6, 7]. The results suggested that vitamin B_12_ status was closely related to the neurodevelopment of young children. Furthermore, compared with the local group, the toddlers in the meat group and the fortified cereal group had higher vitamin B_12_ intake and higher cognitive scores, suggesting that although the vitamin B_12_ intake from the intervention foods in this study was low, the serum vitamin B_12_ level was still improved to some extent, thus improving children’s cognitive levels. In this study, compared to the fortified cereal group, toddlers in the meat group had lower vitamin B_12_ levels but higher cognitive scores. In addition to vitamin B_12_, meat is also rich in zinc and iron, all of which are linked to cognitive development in children. It is therefore necessary to increase the intake of animal-based food in infants in poor areas starting at the age of 6 months. It is also recommended that local cereal be replaced with micronutrient-fortified rice cereal as an initially introduced complementary food.

Serum vitamin B_12_ concentration is the most commonly used marker of vitamin B_12_ status. However, the functional markers tHcy and methylmalonic acid (MMA) have been established as useful indicators of vitamin B_12_ status and may be more sensitive indicators of mild vitamin B_12_ deficiency [6]. In view of the limitations of MMA, such as the high cost of analysis, the need for gas chromatography mass spectrometry, and especially in developing countries, the possibility of concentrations being increased by bacterial overgrowth [17], in this study, both serum vitamin B_12_ and tHcy were selected as the biological basis for the assessment of the vitamin B_12_ status of toddlers. The metabolic conversion of tHcy to methionine is inhibited if the coenzyme methionine synthase is not saturated with vitamin B_12_, leading to the accumulation of tHcy [18]. The results of this study showed that the serum vitamin B_12_ level of young children was negatively correlated with tHcy. However, the percentage of individuals with vitamin B_12_ deficiency screened with criteria of serum tHcy > 12 μmol/L and vitamin B_12_ < 300 pg/mL was 7.8%, significantly lower than that screened by using serum vitamin B_12_ (< 300 pg/mL) alone (34.4%). The cutoff of tHcy which was used to determine vitamin B_12_ deficiency in young children was considered different from that of older children or adults [1]. Therefore, how to better judge the vitamin B_12_ status of infants and young children is worth further study.

In this study, no correlation was found between serum vitamin B_12_ and Hb, MCV, MCH or MCHC levels in toddlers. Animal products are not only the only source of vitamin B_12_ but also the main dietary source of iron. Therefore, inadequate intake of animal-based foods can cause vitamin B_12_ deficiency as well as iron deficiency. As a result, due to the simultaneous deficiency of both, the hematological manifestation induced by vitamin B_12_ deficiency, macrocytic anemia, may not be apparent.

The role of vitamin B_12_ in nucleic acid and protein synthesis determines its effect on the growth and development of infants and young children. Recently, a randomized, controlled double-blind trial found that linear and ponderal growth of North Indian children aged 6 to 35 months improved significantly after vitamin B_12_ supplementation [3]. From the age of 6 months, infants in the study areas were given dietary interventions, such as meat or fortified cereal, but the growth retardation of the local children was still obvious. Our previous studies have found that local children may have subclinical enteritis, induced by frequent exposure to the pathogens found in poor hygienic conditions, which may impair their linear growth [9, 22]. Therefore, more factors should be taken into consideration for improvement of children’s nutritional status in poor rural areas.

There are some limitations in the present study. First, the biomarkers of vitamin B_12_ status were not detected at baseline due to ethical issues, so we did not observe the changes in the aforementioned indexes from 6 to 18 months. Therefore, this study lacks a deep and comprehensive assessment of the effects of various nutritional interventions on vitamin B_12_ status. Second, the study is a subsample nested within a clustered randomized trial, and higher sampling error may exist. In addition, the nonblind design may have generated bias when the subjects were enrolled. These biases may make the research conclusions deviate from the actual situation. Third, the BSID III screening test was used to assess the infants’ development at 18 months of age and was probably the best developmental test for this age group. However, the BSID III had poor predictive ability for intelligence quotient and school achievement later in life. Finally, the determination of serum vitamin B_12_ by chemiluminescence is susceptible to matrix effects and antibody specificity, which may affect the accuracy of detection results.

## Conclusions

Vitamin B_12_ is an essential micronutrient for children’s growth and neurodevelopment, and it only exists in animal products. In poor rural areas of China, vitamin B_12_ deficiency in young children was more common and was associated with low dietary vitamin B_12_ intake. Micronutrient-fortified rice cereal and meat could help improve the nutritional status of vitamin B_12_ in children and might improve their cognitive level.

## Supplementary information

**Additional file 1.** (Nutrition Monitoring of Young Children, Third Version). (PDF 187 kb)

## Data Availability

The datasets used and analyzed during the current study are available from the corresponding author on reasonable request.

## Abbreviations

Hb: Hemoglobin
HcAZ: Head circumference-for-age z-score
LAZ: Length-for-age z-score
MCH: Mean corpuscular hemoglobin
MCHC: Mean corpuscular hemoglobin concentration
MCV: Mean corpuscular volume
MMA: Methylmalonic acid
tHcy: Total homocysteine
WAZ: Weight-for-age z-score
WLZ: Weight-for-length z-score
